# Extraction of soil solution by drainage centrifugation—effects of centrifugal force and time of centrifugation on soil moisture recovery and solute concentration in soil moisture of loess subsoils

**DOI:** 10.1007/s10661-017-5788-7

**Published:** 2017-01-29

**Authors:** Dico Fraters, Gerard J. F. L. Boom, Leo J. M. Boumans, Henk de Weerd, Monique Wolters

**Affiliations:** 1 National Institute for Public Health and the Environment (RIVM), P.O. Box 1, 3720 BA Bilthoven, the Netherlands; 2 TNO Innovation for life, Princetonlaan 6, 3584 CB Utrecht, the Netherlands

**Keywords:** Pore water, Water recovery, Nutrients, Silt loam, Leaching, Centrifuge drainage

## Abstract

The solute concentration in the subsoil beneath the root zone is an important parameter for leaching assessment. Drainage centrifugation is considered a simple and straightforward method of determining soil solution chemistry. Although several studies have been carried out to determine whether this method is robust, hardly any results are available for loess subsoils. To study the effect of centrifugation conditions on soil moisture recovery and solute concentration, we sampled the subsoil (1.5–3.0 m depth) at commercial farms in the loess region of the Netherlands. The effect of time (20, 35, 60, 120 and 240 min) on recovery was studied at two levels of the relative centrifugal force (733 and 6597*g*). The effect of force on recovery was studied by centrifugation for 35 min at 117, 264, 733, 2932, 6597 and 14,191*g*. All soil moisture samples were chemically analysed. This study shows that drainage centrifugation offers a robust, reproducible and standardised way for determining solute concentrations in mobile soil moisture in silt loam subsoils. The centrifugal force, rather than centrifugation time, has a major effect on recovery. The maximum recovery for silt loams at field capacity is about 40%. Concentrations of most solutes are fairly constant with an increasing recovery, as most solutes, including nitrate, did not show a change in concentration with an increasing recovery.

## Introduction

The pollution of groundwater and surface water by agriculture is still one of our major environmental problems. In the USA and Europe, governments implement policies to abate this pollution (Drevno 2016) and, in several European countries, early warning monitoring systems have been set up to follow-up the effect of agricultural measures on leaching from the soil to groundwater and surface waters (Fraters et al. 2011).

Loess soils are agriculturally important soils (Catt 2001), often with groundwater levels at great depth, i.e. an unsaturated zone of more than 10 m in depth. There may, therefore, be a significant lag time between changes in agricultural practises and changes in the (saturated) groundwater quality. In addition, the costs of installing dedicated monitoring wells in these situations are high. Measuring solute concentrations in water leaching from the root zone is an alternative; for examples in China, see Huang et al. (2013), in the USA, Steinheimer et al. (1998) and, in France, Baran et al. (2007). This will provide information about the quality of the water recently leached from the root zone of a field or a farm that is flowing toward groundwater.

There are different methods currently used to study the leaching of solutes in an unsaturated zone, all of which have their advantages and their drawbacks (Fares et al. 2009; Di Bonito et al. 2008; Weihermüller et al. 2007; Schuwirth and Hofmann 2006; Ramos and Kücke 2001; Bufflap and Allen 1995; Litaor 1988). One can distinguish between methods that directly measure leaching and indirect methods that only measure concentration and calculate the amount of leaching. The direct methods measure both the quantity and the quality of the leached water, e.g. with monolith lysimeters (Wang et al. 2012). The quantity of water leached in indirect methods is often calculated with mechanistic models using data from a nearby weather station (Hansen and Eriksen 2016). A further distinction can be made between methods that carry out the measurements in the field and those that limit the measurements to the laboratory; lysimeters and porous cups are examples of the former, drainage centrifugation and batch extraction of the latter. The type of method used will often depend on the monitoring approach. An approach that uses a few monitoring sites in combination with models to assess the effect of the measures will prefer field methods, as repeatedly sampling at the same location with little disturbance is feasible (Grant et al. 2011). An approach based on statistical sampling where a large number of sites are monitored, will prefer indirect, laboratory methods, to extract pore water and to determine its solute concentration, as these methods are less laborious and much cheaper (De Goffau et al. 2012).

The drainage centrifuge method is a well-known method of extracting water from soil samples in order to determine solute concentrations. Briggs (1907; cited by Landa and Nimmo 2003) and Cameron (1911) were the first to use this method in plant nutrition studies. Davies and Davies (1963) were the first to describe and test this method. The method was further developed and tested by Gillman (1976) and Edmunds and Bath (1976). The drainage centrifuge method is used to study processes in soil moisture (Reitzel and Turner 2014; Shand et al. 2000; Bath and Edmunds 1981), the effect of soil storage and sample pre-treatment on solute concentrations (Pérez et al. 2004; Tyler 2000; Chapman et al. 1997; Walworth 1992), the plant availability of solutes (Csillag et al. 1999), fractions of soil moisture (Figueroa-Johnson et al. 2007; Tyler 2000; Giesler et al. 1996) and leaching (De Goffau et al. 2012; Wellings and Bell 1980).

Drainage centrifugation extracts soil moisture by applying a tension that increases quadratically with the centrifugal speed. Centrifugation increases the relevance of the elevation head component of the hydraulic head compared to the suction head by imposing a centripetal acceleration on the soil sample (McCartney 2007). The tension is not homogenous within the soil column. The applied tension developed at a point within the soil column (*P*_*a*_ in cm) can be calculated with (Di Bonito et al. 2008; Edmunds and Bath 1976):

1 $$ {P}_a=\frac{\omega^2}{2\cdot g}\cdot \left({r}_1^2-{r}_2^2\right) $$

with *ω* = angular velocity in radians per second, *g* = acceleration due to gravity in cm·sec^−2^, *r*_1_ = distance from the base of column to the centre of rotation in cm and *r*_2_ = distance from the point of interest within the column to the centre of rotation in centimetres.

The angular velocity (*ω*) is related to the centrifugal speed (*V*_c_ in rounds per minute) with:

2 $$ \omega =2\cdot \pi \cdot \frac{Vc}{60} $$

at equilibrium, *P*_*a*_ will be balanced everywhere by the capillary pressure (N∙m^−2^) (Di Bonito et al. 2008). The extent of soil moisture removal is therefore a function of the centrifuge dimensions and rotation or centrifugal speed, but it is also governed by the weight of sample used, the degree of initial saturation and the material’s pore size distribution (Di Bonito et al. 2008). However, compaction with an increase in dry bulk density and pore size reduction may occur during centrifugation (Jones and Edwards 1993; Edmunds and Bath 1976).

Many studies, however, express the force used to extract the soil moisture as the relative centrifugal force (RCF). The RCF is the ratio between the centripetal acceleration and acceleration caused by gravity (McCartney 2007):

3 $$ RCF=\frac{\omega^2\cdot r}{g} $$

with *r* = distance from a point in the soil column to the centre of rotation in centimetres.

The RCF varies linearly with radius (*r*). This implicates that the RCF increases from the top of the soil column (near the centre of the rotor) to the bottom of the column. In this study, we calculated the RCF for the mid of the soil column.

By sampling the soil below the root zone and extracting soil moisture by drainage centrifugation, the effects of agricultural measures on leaching in loess soils with a thick vadose zone can be monitored. Studies have shown that soil moisture recovery (SMR) from soil samples with drainage centrifugation depends on both the RCF and the time of centrifugation (e.g. Toifl et al. 2003; see Table 1 for more references) and SMR may also depend on soil characteristics, such as soil texture, and soil moisture content (Elkhatib et al. 1987). There is less consensus about the effect of an increase of SMR on measured concentration (Table 1). Some studies showed no or hardly any effect of SMR on solute concentration (Toifl et al. 2003; Reynolds 1984; Gillman 1976), while others showed a significant decrease (Edmunds and Bath 1976) or both increases and decreases depending on the solute and soil (Pérez et al. 2002). However, most of the studies that looked into the specifics of the drainage centrifuge method were limited to the top soil (upper 0.2 m). Studies have rarely looked into processes in soil layers below the root zone, that is, deeper than 1.0 to 1.5 m below the soil surface (Table 1). Studies that have used material from greater depths involved materials with hydrologic and chemical characteristics which are different from loess.

**Table 1:** Overview of set up and results of soil moisture recovery (SMR) studies

Ref.	Soil texture and clay content (cc)	Depth (m)	Treatment and gravimetric soil moisture content (W)	Time and SMR	RCF and SMR	SMR and solute concentration
Present study	Silt loam; cc: 15.6–22.1%	1.5–3.0	None W: 18–25% dry soil	20–240 min 10.5–21.6% at 733*g* 28.1–35.2% at 6597*g*	264–14,191*g* 4.9–34.5% at 35 min	Increase: Ba Decrease: NH_4_, SO_4_ Inconsistent: Cl None: NO_3_, total dissolved C and N, Ca, Cr, Cu, K, Mg, Na, Ni, Sr Below LoD: Al, As, Cd, Fe, P, Pb, Zn
Toifl et al. (2003)	Sandy clay loams	0–0.02	Dried, crushed, sieved and rewet to 42% (W), to 2 and 7.5 kPa, and fresh, field moist (near FC)	5–30 min 23–28% at 220*g* 32–40% at 1982*g*	220–1982*g* 28–40% at 30 min	None: total dissolved P
Pérez et al. (2002)	Clay; cc: 54–67%	0–0.20 and 0.30–0.70	Dried, sieved and rewet to pF 2.5 W: 26–31%	30–60 min 25.6–27.7% at 560*g* 37.8–38.8% at 5080*g*	560–5080*g* 25.6–37.8% at 30 min 27.7–38.8% at 60 min	Increase: NH_4_, NO_3_, Ca, Fe Decrease: Cl, Na, K Non: EC, pH, Al, Mg, Mn, SO_4_, TOC, Zn Below LoD: F
Tyler (2000)	Rendzic Leptosol; cc: low	0–0.10	Sieved (6 mm) and mixed; close to water holding capacity		24–13,350*g* at 60 min 8–67% (almost linear with log RCF)	Sequential extraction Increase: MR-P, P-tot, Mo Decrease: Ca, Mg, Na, Fe, Si Non: Al, K, Mn, S, pH
Grieve (1996)	Organic soil	n.s. (litter layer, Oh horizon)	Mixed and roots removed W: 377% dry soil	5–90 min at 137*g* 14–38%		Decrease: TOC
Walworth (1992)	Loamy sand and sandy loams cc: 22–33%	n.s. (Bt horizon)	None or dried and rewet or frozen (−18 °C) and thawed (4 °C) W: 17.6–34.6%	20–240 min at 750*g* No data on water recovery		(Fresh soil) Increase: EC, Cl, NO_3_, K Inconsistent: pH, Ca, Mg, NH_4_, SO_4_ None: F, Na
Ross and Bartlett (1990)	Coarse-loam Haplorthod	n.s. (Oa and Bhs horizons)	60 days of refrigerated storage		2400 and 9600*g* until enough solution (mostly 45 min) Oa: 26.0–38.0% Bhs: 35.5–47.0%	Increase: pH Decrease: Al None: Cl, F, NO_3_, SO_4_ 390–2400*g* at 30 min (sequential extraction, Oa only) Increase: Cl, F, SO_4_ None: NO_3_
Elkhatib et al. (1987)	Sandy loam to clayey loam; cc: 5.7–20.6%	n.s. (surface)	Dried, sieved and rewet to pF 2.5 (33 kPa) W: 21–63% dry soil	20–60 min. at 49000*g* 41–50% for cc 15.5%, W 58% 56–88% for cc 20.6%, W 37%	12,000–49,000*g* at 20 min 17–31% for cc 15.5%, W 58% 35–58% for cc 10.7%, W 21%	No data
Reynolds (1984)	Silt clay loam and sandy loam	n.s. (Bs horizon)	None W: 43–50% wet soil (FC)	15–240 min 22–36% at 10100*g*	900–10,100 6.6–36.0% at 60 min	None: F
Gillman (1976)	n.s. (Ferrasol and Luvisol) cc: 26–64%	0–0.10	Moistened to pF 2 W: 16–34%	15–60 min (see also right) 2.0–4.2 at 225*g* ^c^ 7.2–11.5 at 900*g*	225^c^–900*g* at 45 min (see also left) 2.7–11.1 for cc 64%, W 34% 3.8–6.6 for cc 26%, W 16%	Fairly constant: EC, Ca, Cl, Mg, K, Na
Edmunds and Bath (1976)	Chalk	30–40 and 490	None	5–140 min 0.07–0.12 ml at 1300 rpm	4220–19,700*g* ^b^ 3.4–90% at 45 min	Decrease: Ca, K, Na None: Mg, Sr
Davies and Davies (1963)	Loam and silt loam	n.s.	None W: 30–47%	15–120 min 33–66% at 1200*g* 41–71% at 2130*g* ^a^	1200–2130*g* ^a^ 50–55% at 30 min 54–67% at 60 min	No data

*n.s.* not specified

^a^RCF for 4000 rpm not provided in paper, estimated with formula [3] and given RCF for 3000 rpm

^b^RCF calculated with formulas [1] and [3] and with *r*_1_ = 11.1 cm and *r*_2_ and *r* = 8.1 cm

^c^RCF for 1000 rpm not provided in paper, estimated with formula [3] and given RCF for 2000 rpm

This research aims to determine whether soil moisture extraction by drainage centrifugation is a robust method for loess samples from below the root zone. Specifically, whether solute concentrations in soil moisture depend on the fraction of soil moisture recovered. We will, therefore, look into the dependency of SMR on both soil characteristics and RCF and time, and we will analyse the relationship between SMR and solute concentration.

## Materials and methods

### Site description

**Fig. 1:**
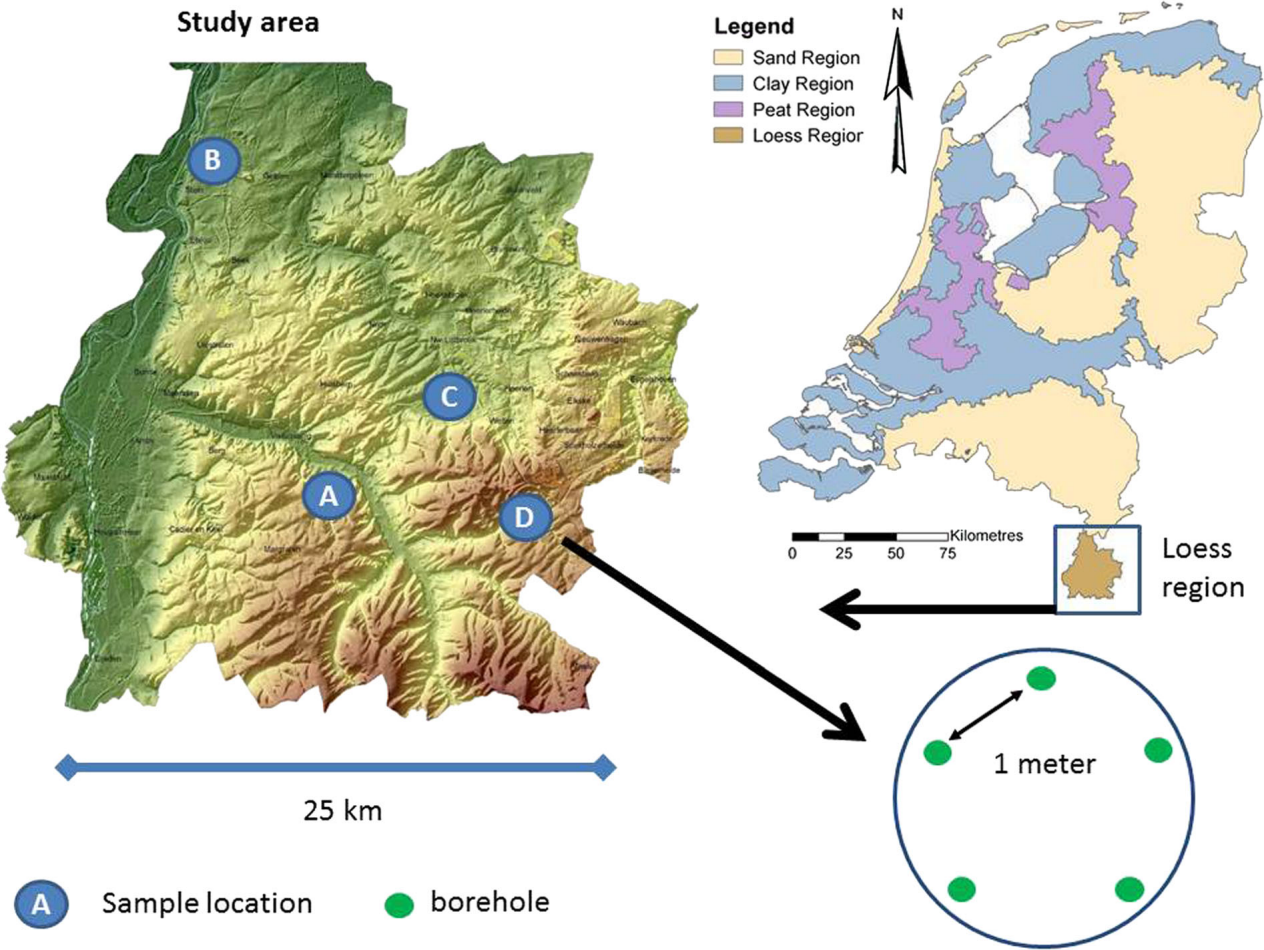
Study area, sample locations (*blue circles*) and boreholes per locations (*green dots in blue circle*). *Brown colours* on left map are high elevations; *green colours* on map are low elevations

The soil samples were taken in the Loess region of the Netherlands, the southern part of the Dutch province of Limburg (see Fig. 1). The landscape in this, about 25 by 25 km^2^, region is slightly undulating (20–325 m above sea level). In the western part of the region is the river plain of the River Meuse, which flows from South to North, and to the East of this river plain, there are plateaus covered with a 2–20-m thick layer of loess (Van Dijk and Kwaad 1996). Soils are mainly Eutric-Gleyic Fluvisol in the river plain and Haplic Luvisols outside the river plain (European Commission 2005). The climate is temperate maritime. Annual precipitation increases from 750 mm in the northwestern part in this region to 950 mm in the southeast (data Royal Netherlands Meteorological Institute, average 1981–2010).

Four locations (A–D) were selected at four different commercial farms (Fig. 1); one location was in a grassland field on a dairy farm, and three locations were in arable fields on arable farms. Locations were selected at farms participating in the Minerals Policy Monitoring Programme (De Goffau et al. 2012). Main selection criteria were that locations had at least a 3-m thick layer of loess and a soil moisture nitrate concentration of more than 50 mg/l in the subsoil at previous samplings (Fraters et al. 2016). Other selection criteria were a good geographical distribution of locations within the Loess region, locations at different farm types (dairy and arable) and at different positions in the landscape. Location A was in an arable field located at 125 m above sea level (SL) near the centre of a SW-NE directed dry valley (80–165 m + SL). Location B was in an arable field at 30 m + SL in a flat area near the River Meuse. Location C was in an arable field at 90 m + SL in a flat area on a lower plateau. Location D was in a permanent grassland field at 175 m + SL on a gentle slope (2°).

### Sampling

The samplings were carried out in accordance with the procedure used in the Minerals Policy Monitoring Programme (De Goffau et al. 2012) between 30 June 2014 and 3 July 2014. During samplings, the sky was cloudy and the temperature between 18 and 21 °C; it was mostly dry, however, some rainfall occurred in the form of short showers. Sampling was interrupted during the showers, and the boreholes were covered. Five boreholes in each field were drilled by hand using an Edelman auger. Boreholes were located at the points of an imaginary pentagram with a distance between points of about 1 m (Fig. 1). The upper 0.3 m of soil was drilled with an auger of 0.10 m in diameter. Then a PVC ground sleeve was placed into the borehole to prevent contamination with topsoil material. Drilling was continued with an auger of 0.07 m in diameter until 1.5 m depth. Thereafter, the soil layer between 1.5 and 3.0 m was sampled in steps of 0.1 m with the same auger. A clean core was retrieved of each 0.1 m by removing the soil from the flanks and the top and bottom of the core with a knife. Each of the 15 soil cores per borehole was stored separately in a firmly closed plastic box.

**Fig. 2:**
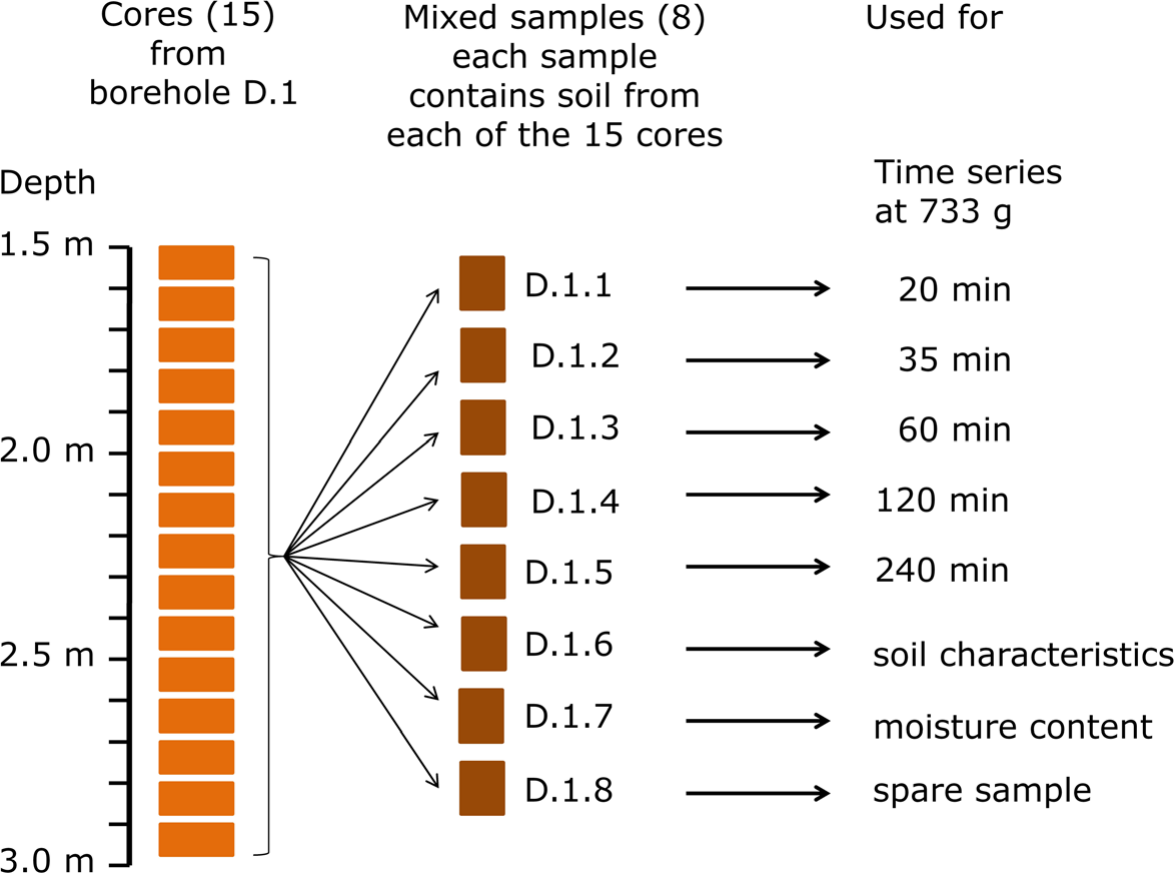
Schematic representations of preparation of mixed samples and use of individual samples for tests and analysis of soil characteristics and soil moisture content

The 15 soil cores of each borehole were divided over eight 800-ml glass containers which each were placed on a weighing scale (Fig. 2). This procedure was carried out inside the back of a truck near the field. In this way, eight replicate mixed samples per borehole were prepared directly after finishing the borehole. This led to four locations times five boreholes times eight mixed samples. The mixed samples were coded to location ID–borehole number–mixed sample number, e.g. A.3.4. One mixed sample per borehole, the one used for soil characterisation, was conveyed in a special sac used by BLGG AgroXpertus for storage and transport. All mixed samples were stored in a dark and cool environment until processing. This procedure minimises evaporation. Each mixed sample weighed about 500 g.

### Centrifugation time and speed

Glass containers with mixed soil samples were removed from the refrigerator and stored at room temperature in the dark for at least 12 h before processing. Each mixed sample was split into two subsamples of equal weight by filling two centrifuge apparatuses. Centrifugation was carried out at 25 °C (according to internal procedure AC-W-016). After centrifugation, the collected soil moisture of the two subsamples was mixed and filtered with a 0.45-μm polyethersulfone (PES) syringe filter (Dispolab) using a polypropylene (PP) syringe.

Centrifugation of each mixed sample was performed, as described by Aitken and Outhwaite (1987), using a Sorval RC6+ centrifuge with a fixed angle (23°) SLA-3000 rotor with six 500-ml positions. The centrifuge apparatus was entirely made from Delrin (polyoxymethyleen) (Giesler and Lundstrom 1993). The soil sample compartment had an inner diameter of 0.053 m and a length of 0.115 m, and the cup for collecting soil moisture had the same inner diameter and a length of 0.015 m. A Sartorius FT-2-205-58058 filter paper was placed on the perforated base of the soil sample compartment.

We used samples from two boreholes from each of the three locations A, B and C to study the effect of centrifugation time (20, 35, 60, 120 and 240 min) at two RCFs (borehole 1 for 733*g* and borehole 2 for 6597*g*); see Table 2. The samples of borehole 1 from location D were used for preliminary tests on SMR at different RCFs, and the time of centrifugation and those of borehole 2 were stored. We used samples from three boreholes (numbers 3, 4, and 5) from each of all four locations to study the effect of RCF. Time of centrifugation in these experiments was fixed at 35 min. RCF levels were 117 (location A only), 264, 733, 2932 (except location A), 6597 and 14,191*g*. The centrifugal speed in rounds per minute was 1000, 1500, 2500, 5000, 7500 and 11,000 rpm. The weight of fresh soil and soil after centrifugation were recorded to calculate soil moisture yield. SMR, also called water recovery or centrifugal drainage efficiency, was calculated by dividing the soil moisture yield by the soil moisture content (SMC). We used a separate sample for each combination of time and RCF. To study the effect of RCF on solute concentration, the centrifugal extracts of mixed samples from the three boreholes per location (numbers 3, 4 and 5) for each RCF level were mixed in order to have enough soil moisture to do additional chemical analysis (see section 2.4). For example, the extracts of samples D.3.1, D.4.1 and D.5.1 (see Fig. 2 for numbering)—all centrifuged at 264*g* for 35 min—were mixed before chemical analysis.

**Table 2:** Use of mixed samples in experiments

Location	Borehole	Mixed samples	Use
A	A.1	A.1.1–A.1.5	Time series (20–240 min) at 733*g*
	A.2	A.2.1–A.2.5	Time series (20–240 min) at 6597*g*
	A.3–A.5	A.3.1, .4.1, .5.1–A.3.5, .4.5, .5.5	RCF series^a^ (117–14,191*g*) at 35 min
B	B.1	B.1.1–B.1.5	Time series (20–240 min) at 733*g*
	B.2	B.2.1–B.2.5	Time series (20–240 min) at 6597*g*
	B.3–B.5	B.3.1, .4.1, .5.1–B.3.5, .4.5, .5.5	RCF series^a^ (117–14,191*g*) at 35 min
C	C.1	C.1.1–C.1.5	Time series (20–240 min) at 733*g*
	C.2	C.2.1–C.2.5	Time series (20–240 min) at 6597*g*
	C.3–C.5	C.3.1, .4.1, .5.1–C.3.5, .4.5, .5.5	RCF series^a^ (117–14,191*g*) at 35 min
D	D.1	D.1.1–D.1.5	Preliminary time and RCF tests
	D.2	D.2.1–D.2.5	Stored
	D.3–D.5	D.3.1, .4.1, .5.1–D.3.5, .4.5, .5.5	RCF series^a^ (117–14,191*g*) at 35 min

^a^SMR is determined for each of the five mixed samples per borehole; concentrations are measured in combined extracts of three mixed samples with same treatment from x.3, x.4 and x.5

### Physico-chemical analysis

Standard soil analyses were carried out on one of the eight mixed samples from each borehole (Fig. 2) by BLGG AgroXpertus according to Dutch or international standard procedures: soil texture (NEN 5753; sand (>50 μm) gravimetric, clay (<2 μm) density measurement using Stokes formula, silt calculated), organic matter (NEN 5754, weight loss at 550 °C), inorganic C (ISO 10694, 1000 °C with infra-red spectro-photometry), cation exchange capacity (CEC) (ISO 23470, extraction with 0.0166 M cobalt hexamine tri-chloride), pH (NEN-ISO 10390, 1:10 *v*/*v* 0.1 M KCL_2_ and 1:10 *v*/*v* water suspension). SMC (g of water per 100 g dry soil) was determined on a separate mixed sample by weight loss at 105 °C in the laboratory by TNO.

Centrifuge extracts from the time series samples were analysed for nitrate, chloride and sulphate (ion chromatography; internal procedure AC-W-066), and for ammonium (acidification with H_2_SO_4_, pH<2; spectrophotometry/continuous flow analysis; internal procedure AC-W-027). Centrifuge extracts in the speed series were additionally analysed for dissolved organic carbon (infrared), dissolved total N (spectrophotometry/continuous flow analysis; internal procedure AC-W-024) and molybdate reactive phosphorus (spectrophotometry/continuous flow analysis; internal procedure AC-W-023), and for dissolved total P and cations (ICP-MS; internal procedure AC-W-036). These are the default chemical analysis for the Minerals Policy Monitoring Programme (De Goffau et al. 2012).

As part of this study, a test was carried out with MilliQ water that showed that the materials used in the extraction and cleaning of the soil moisture (centrifuge apparatus, filters, tubes) may release DOC at about 2.4 mg/l (factor 8 above limit of detection), and also ammonium (0.15 mg/L), copper (1.5 μg/L) and strontium (2.4 μg/L) at a factor 2 above the limit of detection. All the other elements were below the limit of detection.

### Data analysis

Data handling and statistical analysis were performed with R (version 3.1.0) (R Core Team 2015) using R studio (version 0.98.507) for Windows 7.

The relationship between SMR and RCF—using data from time and speed experiments, but with a centrifugation time of 35 min—was fitted with a Langmuir type of formula (see Fig. 4)

4 $$ SMR=\frac{SMR_{\max}\cdot {K}_a\cdot RCF}{1+{K}_a\cdot RCF} $$

with *SMR*_max_ as the maximum percentage of the total water that can be removed during 35 min, *K*_a_ as the constant and *RCF* as the average relative centrifugal force. The R *nls* command was used to calculate nonlinear least squares and to perform curve fitting.

A restricted maximum likelihood (REML) analysis was carried out to study the effect of soil parameters (Table 3), speed (RCF) and time of centrifugation (as fixed effects), and of location (as random effect) on water yield. The R *lmer* command was used with REML = TRUE. For this analysis, the results of the samples from the different boreholes with the same treatment were considered as repetitions per location. The grid shown in Fig. 5 is based on a simplified linear regression analysis (*lm* command) considering all 20 boreholes as independent observations.

**Table 3:** Soil characteristics of subsoil (1.5–3.0 m depth) at sample locations (averages of five samples per location)

Parameter	Location A	Location B	Location C	Location D
Texture (FAO)	Silt loam	Silt loam	Silt loam	Silt loam
Clay (﻿%﻿ <2 μm)	22.1a	15.6b	19.2c	21.9a
Silt (%﻿ 2–63 μm)	74.1ab	71.8b	77.6a	75.6a
Sand (% 63–2000 μm)	4.0a	12.7b	3.4a	2.5
Organic matter (%)	1.88a	2.06b	1.72c	1.84ac
Inorganic C (%)	0.01a	0.45b	0.72c	0.03a
CEC (mmol/kg)	120a	102b	115a	109ab
pH-H_2_O	8.16a	8.52b	8.58b	7.46c
pH-KCl	6.88a	7.72b	7.64b	6.14c
SMC^a^ (%)	18.1a	25.1b	23.3c	21.0d

Values in the same row followed by the same letter are not significantly different by Turkey’s multiple comparisons test (*P* > 0.05)

^a^Soil moisture content in grammes per 100g of dry soil (105 °C)

An analysis of variance (ANOVA) was carried out to study the effect of SMR, location and their interaction on solute concentration. The R *anova* command was used in combination with the *lm* command. For this analysis, all results per location were treated as if they were independent observations.

## Results

### SMR and yield

**Fig. 3:**
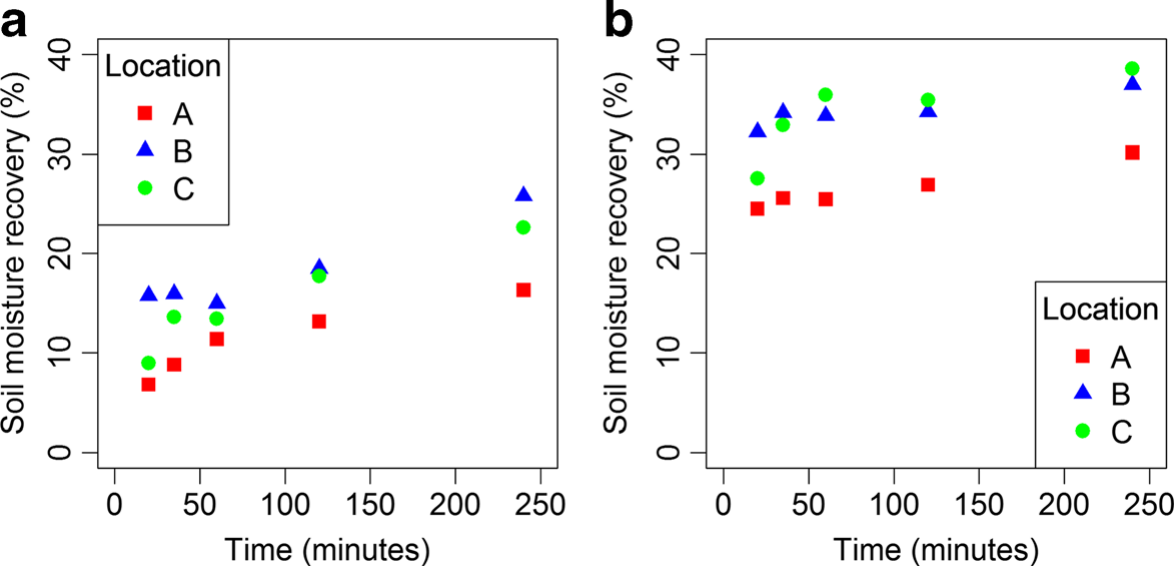
Soil moisture recovery (% of soil moisture content) as a function of time of centrifugation at **a** RCF of 733*g* and at **b** RCF of 6597*g*

**Fig. 4:**
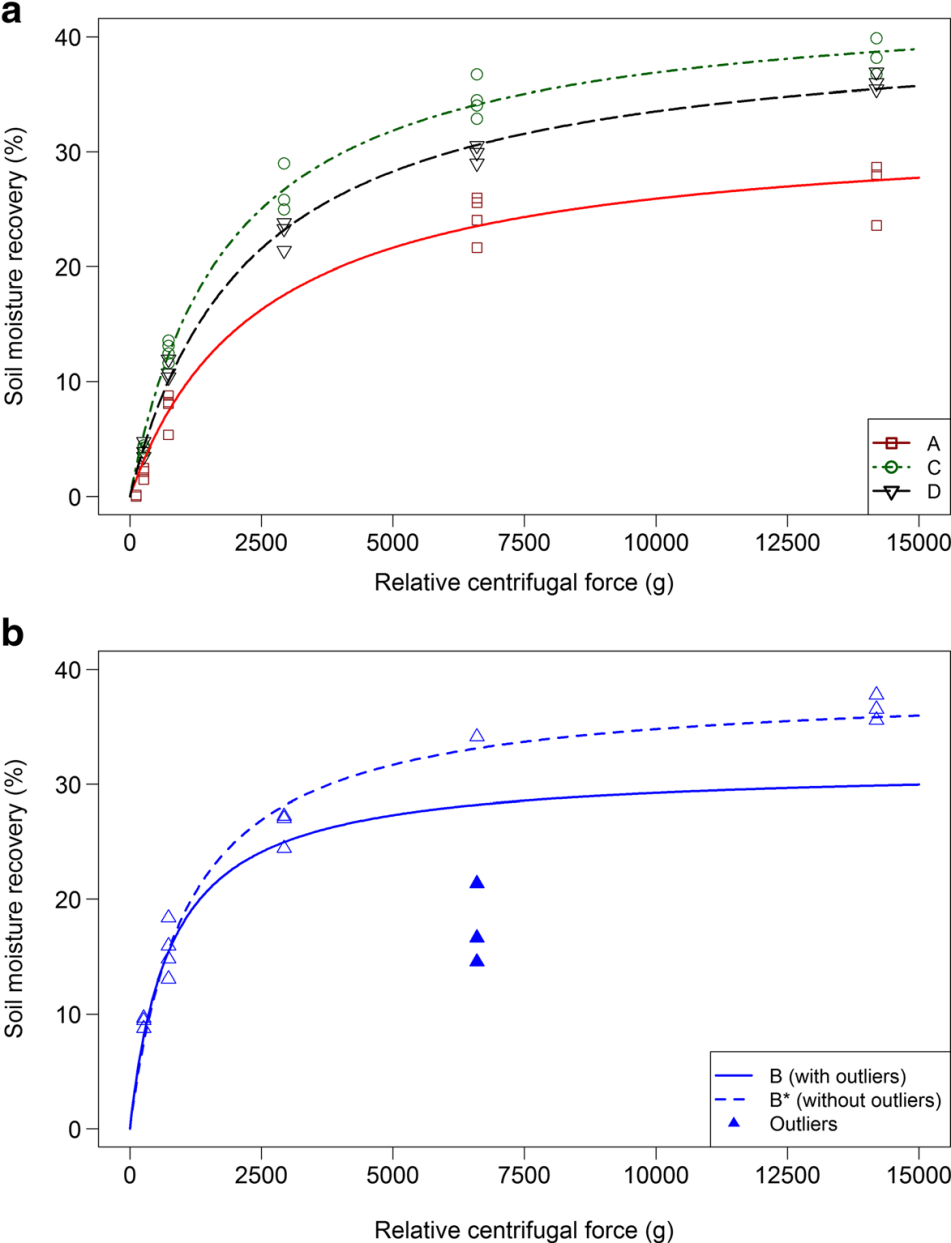
Soil moisture recovery (%) as a function of speed, expressed as relative centrifugal force (RCF), after 35 min of centrifugation (values for all available boreholes per location). Figure 4a for location A, C and D and Fig. 4b for location B. *Lines* show best fit of Langmuir parameters (see Table 2). *Dashed line* in Fig. 4b shows fit for location B without data from the speed experiment at RCF = 6597*g* (B*)

SMR increased with both time (Fig. 3) and RCF (Fig. 4). Maximum measured SMRs ranged from 26.8 up to 38.3% when centrifuging at 14191*g* for 35 min (see Table 4 and Fig. 4). Maximum measured SMRs were only slightly higher when centrifuging for 240 min at 6597*g* (30.2–38.6%; Fig. 3).

**Table 4:** Langmuir parameters defining the relationship between soil moisture recovery (SMR %) and relative centrifugal force (RCF *g*) at 35 min, and measured SMR at RCF of 14,191*g* for 35 min (SMR_max_, measured)

Parameter	Location A	Location B	Location B*	Location C	Location D
SMR_max_^a^, measured	26.8a	36.7b	36.7b	38.3b	36.1b
SMR_max/c_, estimated	32.3	31.5	38.3	43.8	41.1
Constant (Ka)	4.06 10^−4^	13.06 10^−4^	9.277 10^−4^	5.329 10^−4^	4.405 10^−4^
Std error WR_max/c_	1.63	2.98	1.36	1.05	0.91
Std error Ka	0.731 10^−4^	5.337 10^−4^	1.107 10^−4^	0.444 10^−4^	0.324 10^−4^

B* dataset for location B without data for RCF = 6597*g* at boreholes B.3 till B.5 (see Fig. 4)

^a^Values in the same row followed by the same letter are not significantly different by Turkey’s ﻿mul﻿tiple compariso﻿ns﻿ test (*p* > 0.05)

The effect of time on SMR is larger at low RCFs (733*g*) than at high RCFs (6597*g*) (Fig. 3). At low RCFs, SMR increased by 71% between 35 and 240 min and at high RCFs by 15%. SMR increased by 9–21% when RCF was increased from 6597 to 14,191*g* at 35 min (Fig. 4). Only at location B, was the increase in SMR between 6597 and 14,191*g* much larger. This was probably due to the deviating low SMR (average 18%) at 6597*g* in the RCF experiment (Fig. 4b). In the time experiment, the SMR at the same speed and time was 34%. This SMR of 34% at 6597*g* in the time experiment is more in line with the SMRs measured at lower and higher RCFs in the RCF experiment (see fitted line B* in Fig 4b).

The absolute maximum percentages of removable soil moisture were estimated using formula [4] and the data from the RCF experiments (centrifugation time 35 min). The estimated maxima ranged from 32 to 44%. Estimated maxima for SMR were roughly 15% higher than the measured maxima (Table 4).

**Fig. 5:**
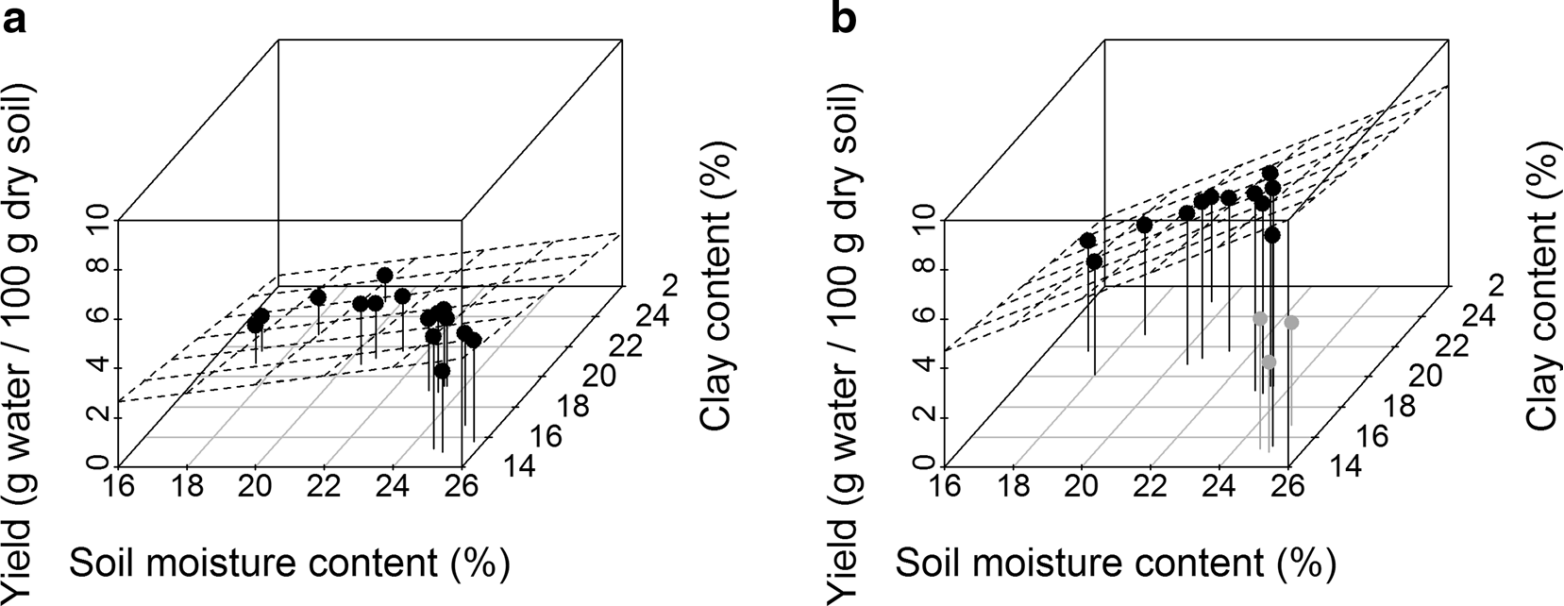
Relationship between yield (grammes of water per 100 g of dry soil) and soil moisture content (SMC) and clay content (%) at a low **a** and a high RCF **b** shown as grid. Three samples in *grey* are samples with deviating yields. These *grey dots* are not used to calculate the relationship between yield and SMC and clay content shown as grid

Soil moisture yield, expressed as grammes of water extracted per 100 g of dry soil, was higher when the SMC was higher, but the yield was lower when the clay content was higher (Table 5 and Fig. 5). The effect of clay was about 0.2 g of water per 100 g of dry soil per %-point of clay and did not differ significantly between RCF levels. The effect of SMC was greater at higher RCFs; 0.17 g per %-point at 733*g* and 0.37 g per %-point at 6597*g* (Table 5). Other soil parameters (Table 3) did not have a significant effect on yield (*t* value < 2; data not shown).

**Table 5:** Estimated effect of soil moisture content (SMC in %) and clay content (%) on yield (grammes water/100g dry soil) for low and high RCF (with location as random effect). Three samples with deviating yields (see Fig. 4b) were not used to calculate the relationship

RCF (g)	Fixed effects	Estimate	Std. error	*t* value
Low (733)	(Intercepts)	2.51	2.10	1.20
	SMC	0.172	0.053	3.24
	Clay content	−0.185	0.052	−3.54
High (6597)	(Intercepts)	2.63	2.71	0.97
	SMC	0.375	0.099	3.78
	Clay content	−0.204	0.070	−2.91

### Solute concentration

**Fig. 6:**
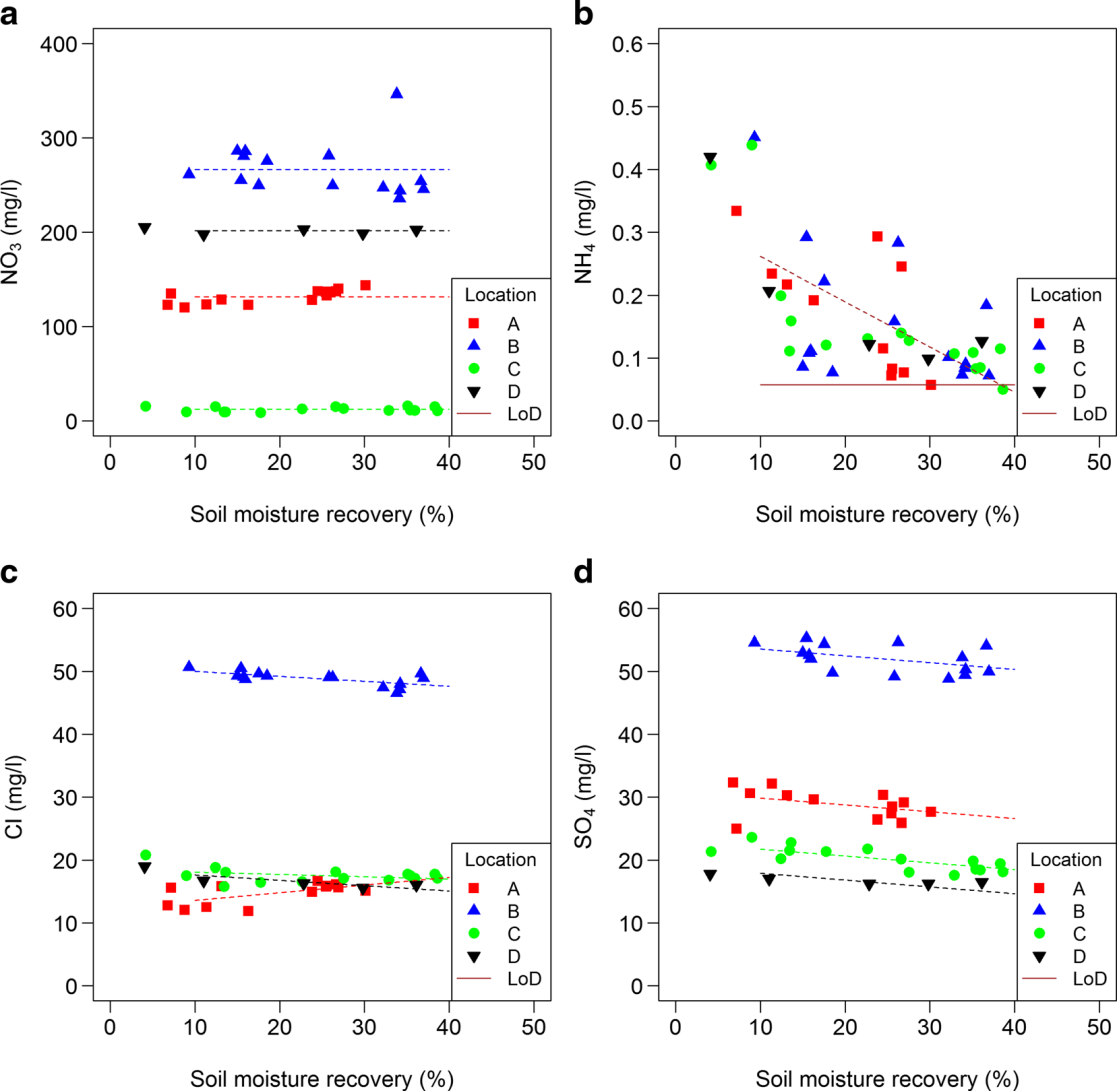
Relationship between solute concentrations (a: NO_3_, b: NH_4_, c: Cl and d: SO_4_) in extracts and soil moisture recovery (% of the total soil moisture); see Table 4 for specifications. Limit of detection (LoD) is shown when relevant

Nitrate concentrations differed significantly between locations and ranged between 10 and 350 mg/l (Table 6, Fig. 6). Concentrations were not dependant on SMR within locations with SMRs ranging between 5 and 40% of SMC. Also, chloride and sulphate concentrations differed between locations. Sulphate showed a decrease in concentration with an increase in SMR (−3.8 mg/L between 5 and 40% SMR). For chloride, at location A, a reversed trend was found, which is significantly different from the trends at other locations. For sulphate, there was no significant difference in trends between locations. Ammonium concentration also showed a decrease with an increase in SMR (−0.25 mg/L between 5 and 40% SMR). The ammonium concentration did not differ between locations, nor did the trends.

**Table 6:** Significance (*p* value) and direction of effect (if *p* value < 0.05) of soil moisture recovery (SMR) and significance of location and interaction between locations and SMR on solute concentration in extract

Parameter	SMR	Locations	Locations: SMR
Ba	+/0.02	<0.001	0.68
Ca	0.25	<0.001	0.59
Cl	0.12	<0.001	<0.001
Cr	0.17	<0.001	0.27
Cu	0.40	0.42	0.92
DOC	0.22	0.23	0.47
K	0.26	0.64	0.87
Mg	0.30	<0.001	0.83
Na	0.71	<0.001	0.49
N total	0.43	<0.001	0.86
N NO_3_	0.97	<0.001	0.49
N NH_4_	−/<0.001	0.98	0.84
Ni	0.71	0.06 (.)	0.41
SO_4_	−/<0.001	<0.001	0.76
Sr	0.72	<0.001	0.95
Zn	0.52	0.17	0.23

**Fig. 7:**
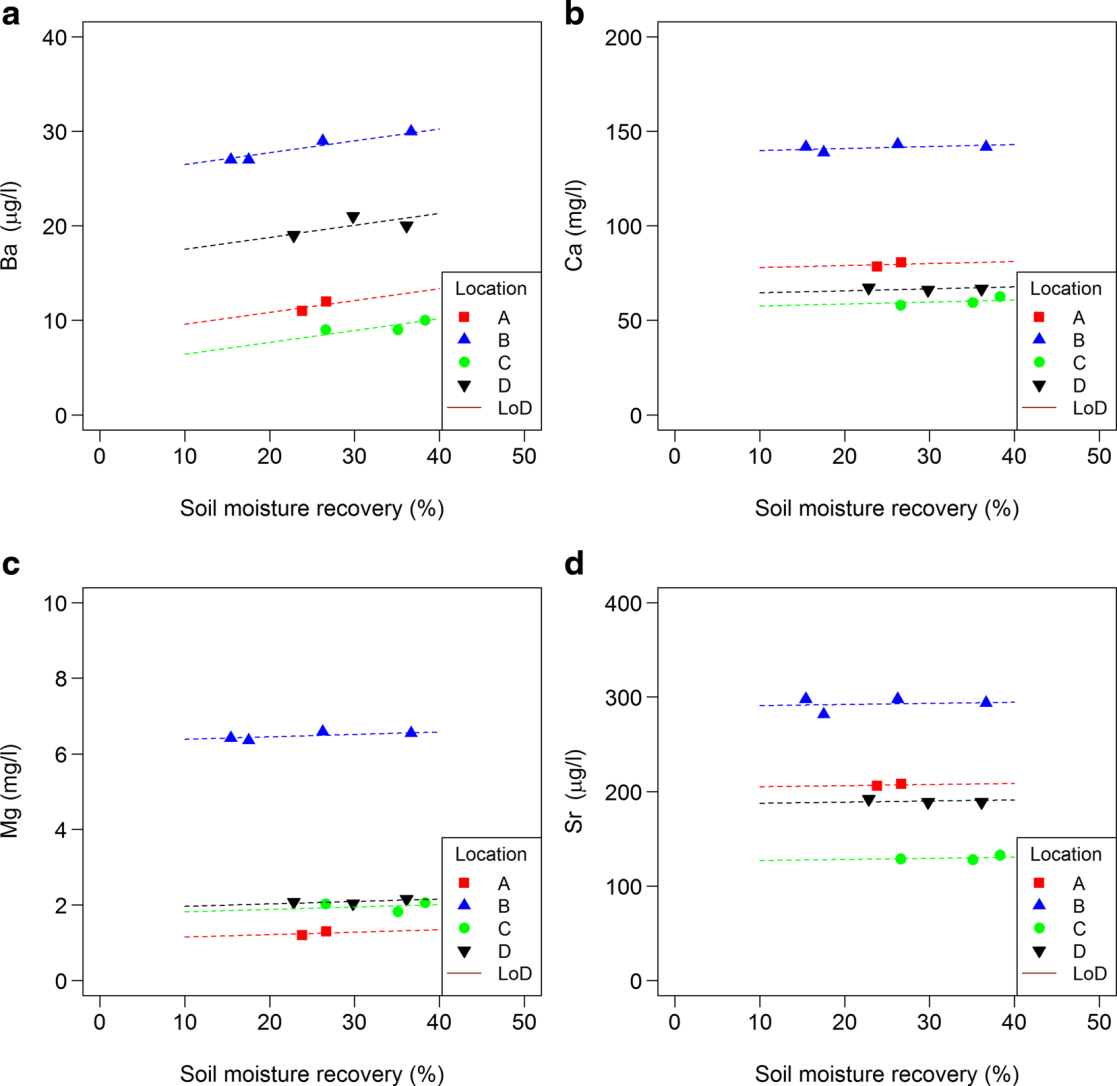
Relationship between solute concentrations (a: Ba, b: Ca, c: Mg and d: Sr) in extracts and soil moisture recovery (% of the total soil moisture); see Table 4 for specifications. Limit of detection (LoD) is shown when relevant

**Fig. 8:**
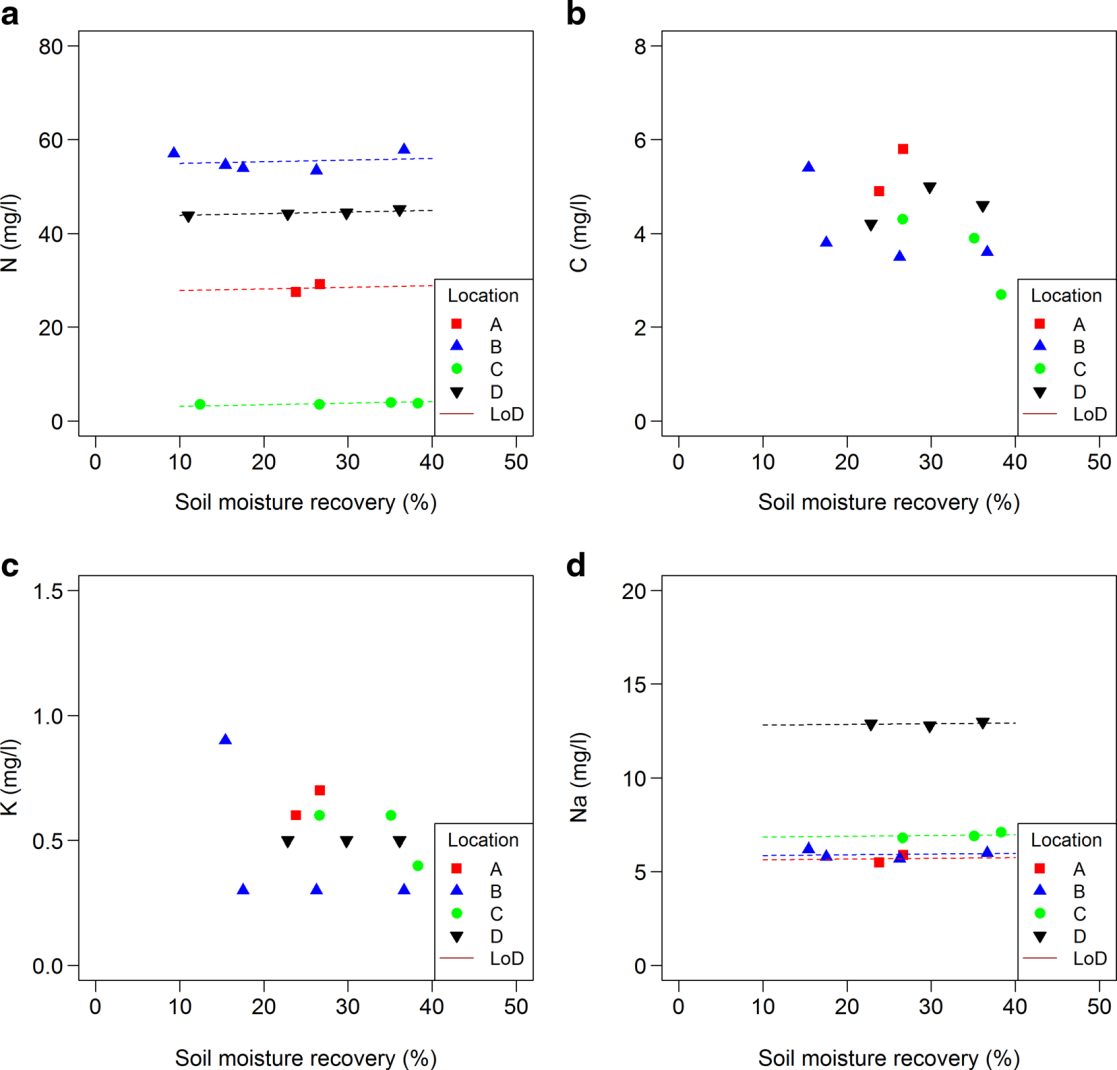
Relationship between solute concentrations (a: total dissolved N, b: DOC, c: K and d: Na) in extracts and soil moisture recovery (% of the total soil moisture); see Table 4 for specifications. Limit of detection (LoD) is shown when relevant

**Fig. 9:**
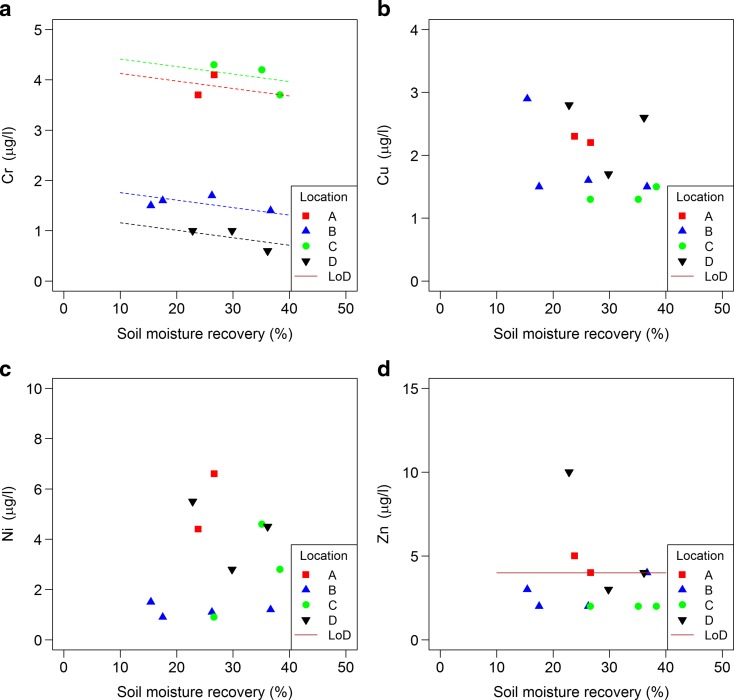
Relationship between solute concentrations (a: Cr, b: Cu, c: Ni, d: Zn) in extracts and soil moisture recover (% of the total soil moisture); see Table 4 for specifications. Limit of detection (LoD) is shown when relevant

For other solutes, fewer results were available for statistical analysis as there were no data from the time series. Only Ba showed a significant increase in concentration with an increasing SMR (Fig. 7; 4.4 μg/L between 5 and 40% SMR). For other solutes, no significant trend was detected. Concentrations for Al, As, Cd, Fe, Mn, ortho P, total dissolved P, Pb and Zn were all around the limit of detection. Concentrations of total dissolved N, Ba, Ca, Cr, Mg, Na and Sr differed between at least two locations. For Cu, DOC, K and Ni no significant differences in concentration were found between locations (Figs. 8 and 9).

## Discussion

### SMR and yield

SMRs of about 27–38% at about 6600*g* for silt-loam subsoil at field capacity, as found in this study, are in line with the results reported by Reynolds (1984) for Bhs-horizons of loamy soils (SMR of 36% at 10100*g*). Other studies also show that the drainage centrifugation method only recovers a proportion of water present in the soil (Table 1).

Positive effects of time and RCF of centrifugation on SMR were reported by others as well (Davies and Davies 1963; Edmunds and Bath 1976; Gillman 1976; Reynolds 1984; Elkhatib et al. 1987; Pérez et al. 2002; Toifl et al. 2003) (Table 1). The results of this study show that RCF rather than centrifugation time has a major effect on SMR. This is in accordance with findings of Toifl et al. (2003) for experiments with soils that were dried, rewet and equilibrated for at least 6 h. They reported that they had had similar results for SMR for field moist soils. For soils that had not been equilibrated after rewetting, time was more important than RCF.

In this study, SMR increased with centrifugation time up to 240 min, with a larger increase at a low RCF (733*g*) than at a high RCF (6597*g*). Reynolds (1984) found, at a higher RCF (10,100*g*) than we used, that centrifugation time in excess of an hour produced no increase in the amount of water removed. This confirms that time becomes less important at higher RCFs. Data from Davies and Davies (1963), also using loamy soils, showed no clear increase after 90 min at an RCF of 2130*g*. However, SMR still increased with time up to 120 min (maximum time) at 1200*g*. This difference in the effect of time on SMR with this study might be due to a difference in packing of the samples. Davies and Davies (1963) ‘tamped down’ the soil before centrifugation; they also used the same sample for their time experiment (sequential extraction). In this study, samples were loosely packed, and we used a different sample for each time level (parallel extraction).

The dependency of SMR on SMC and clay content, found in this study, was also reported by others. Higher SMCs resulted in higher recoveries of soil moisture in the study completed by Davies and Davies (1963), Jones and Edwards (1993), and Toifl et al. (2003). Higher clay contents resulted in lower SMRs in the research carried out by Elkhatib et al. (1987) and Adams et al. (1980), and Reynolds (1984) found a lower SMR for a silt clay loam than for a sandy loam. SMR will depend on the soil matric potential, i.e. the force with which water is held within the soil. Soil type, clay content and SMC are well-known factors influencing the soil matric potential (e.g. Matula et al. 2007). However, Gillman (1976) found no apparent relationship between the percentages of water removed and the clay or carbon content. He attributed that to the arbitrary cut-off time of 45 min centrifuge time that had been chosen. Both Gillman (1976) and Elkhatib et al. (1987) found a larger increase in SMR over time from finer textured soils than from coarser textured soils. This might be due to a slower moisture transport in fine textured soils. So, although most of the removable soil moisture was extracted from coarser textured soils in 45 min, more soil moisture will be able to be extracted from finer textures soils after 45 min.

### Solute concentration

Most solutes did not show a change in concentrations with SMR increasing from 5 up to 40%. Only Ba showed an increase in concentration (Fig. 7), and SO_4_ and NH_4_ showed a decrease (Fig. 6) with an increase in SMR. However, the measured NH_4_ concentrations, as well as Cu and DOC concentrations, are in a range which indicates that they may have been influenced by the release of these substances by materials used in the experiments (see Section 2.4); therefore, the results are not conclusive for these solutes. Also some Sr was released by materials used in the experiments, but measured concentrations are at least a factor 50 higher. On average, there was no effect on Cl, but trends in Cl with an increase in SMR differed between locations (Fig. 6). Location A, the only location with a trend (upward), had the highest clay content and the lowest SMC (Table 3). It is not clear how this could explain a higher chloride concentration in soil moisture extracted from smaller pores. In this study, effects on Al, As, Cd, Fe, Mn, ortho P, total dissolved P, Pb and Zn concentrations could not be detected as all concentrations were around the limit of detection.

Several studies also reported that there was no relationship between solute concentration and SMR. Toifl et al. (2003) found that the total dissolved P in two sandy clay loams was not affected by SMR in the range of 23–40%, however, it was effected by soil type, equilibration time after rewetting the dried soil and soil moisture content. Dissolved P concentrations in the present study were below the limit of detection. Reynolds (1984) referred to a preliminary study that revealed no differences in fluoride concentration with yield, however, fluoride was not measured in the present study. Gillman (1976) reported that successive increments of soil moisture were reasonably constant in soil moisture composition, i.e. electrical conductivity (EC), Ca, Mg, K, Na and Cl, for SMRs between 4% and 27% for a Red Podzolic (31% clay) and between 22% and 100% for a Yellow Earth (10% clay). This is in accordance with the results of the present study carried out with soils with a clay content in between the soils used by Gillman (1976) (15.6–22.1%) and with similar ranges of SMRs (5–35%).

On the other hand, there are also studies that did find a relationship between solute concentration and SMR. In chalk sediments, Edmunds and Bath (1976) found significant, but small decreases for Ca, K and Na with SMR increasing from 20 up to 90% (most observations had a lowest SMR of 40%); no effects were found for Mg and Sr which is similar to results of this study. Also Tyler (2000) found decreases for Ca and Na, as well as for Fe and Si, with increases in SMR of 8–67% for a calcareous soil with a low clay content. Contrary to Edmunds and Bath (1976), Tyler (2000) found a decrease for Mg as well. Tyler (2000) attributed the decrease to lower concentrations of these elements in the micropores. In the studies of Tyler (2000) and Edmunds and Bath (1976), the maximum SMRs are much higher, and the ranges between minimum and maximum SMR was much larger than in the present study. This might, at least, partly explain why the present study did not detect decreases for these elements. In addition, Tyler (2000) carried out a sequential extraction, while in the present study and in the study of Edmunds and Bath (1976), parallel extractions were performed. High SMR extracts of parallel extractions always are a mixture of soil moisture originating from both micropores and bulk solution. Pérez et al. (2002) found that SMR significantly influenced the composition of the extracted soil moisture from clay soil samples from different depths (soil horizons), even though the ranges between minimum and maximum SMR were smaller than in the present study (average SMRs were between 25 and 39%). However, the results were not the same for all depths. Like Edmunds and Bath (1976), Pérez et al. (2002) found decreasing concentrations of K (3 out of 4 depths), and Na (1 out of 4 depths) with an increasing SMR, but increasing Ca concentrations (2 out of 4 depths). There was no significant trend for NO_3_, which was similar to the results of this study, and none for SO_4_, which was different to the present results. For 2 out of 4 depths, a decrease in C1 was found and an increase in NH_4_ (not at the same two depth levels).

There are several difficulties with interpreting the results of the present study compared to the result of other studies:

- Firstly, concentrations may show a different trend when an organic-rich topsoil is studied (used in many studies) rather than an organic-poor subsoil, which was used in this study. For example, data from several studies showed that nitrate concentrations in soil moisture differed between extraction methods that sample different fractions of soil moisture. These differences in nitrate concentrations between methods varied with depth and thereby also with organic matter content (Ross and Bartlett 1990; Djurhuus and Jacobsen 1995; Ranger et al. 2001).
- Secondly, there may have been an effect of soil pre-treatment on the results reported by others. Walworth (1992) stated that neither drying-rewetting nor freezing-thawing provided a good means of storing soil samples prior to soil solution extraction. Jones and Edwards (1993) concluded that soil moisture should be separated from field moist soil as quickly as possible after sample collection when they compared drainage centrifugation extract from field moist, wetted-up field moist and rewetted air-dry soils. Van Erp et al. (2001) showed that time and manner of drying soil samples had a significant effect on solute concentration in a batch experiment.
- Thirdly, it is not always clear which fraction of soil moisture is considered in each study, as both the ranges of RCFs used to extract soil moisture and the initial SMCs varied greatly between studies (Table 1). Even differences in the length of the soil samples (length of the tube used in the centrifuge) between studies may have had an effect, as soil moisture content does not differ linearly within the soil sample after centrifugation (Jones and Edwards 1993). This is due to the non-linear force field within the sample (Di Bonito et al. 2008). If the element concentration in micropores differs from the concentration in the bulk solution, as suggested by Tyler (2000), then changes in concentration with increasing RCFs will only be detected when micropore water is extracted at higher RCFs.

In regards to the extracted soil moisture fraction, this study roughly extracted the fraction between pF 2.5 and pF 3.6; the relatively mobile and plant-available water. This range was calculated using measured SMCs (Table 3) and SMRs (Figs. 3–4), and the soil water characteristics and bulk densities provided by Wösten et al. (2001) for a silt loam subsoil in the Netherlands (see Table 4, subsoil O15, and the formula on page 14 in Wösten et al. 2001). The lower boundary of pF 2.5 concerned field capacity. The upper boundary of pF 3.6 is lower than the average pF of 4.6 applied with an RCF of 11,000*g* (Formula [1]). The difference between these two values might be due to capillary breaks—the soil sample is a collection of small fresh clods and not an undisturbed or a well-homogenised (dried, sieved and rewet) soil sample. Compaction that occurs during centrifugation and which gives rise to pore size reduction (Jones and Edwards 1993; Edmunds and Bath 1976) does not influence the soil water retention curve unless near saturation (McCartney 2007). The lesser effect of time on SMR at higher RCFs compared to lower RCFs might perhaps be due to a faster and stronger compaction of soil at high RCFs, which increases the connections between clods and thereby water flow.

## Conclusions

This study shows that drainage centrifugation offers a robust, reproducible and standardised way of determining solute concentrations in mobile soil moisture in silt loam subsoils. Loess soils are agriculturally important soils, often with groundwater levels at a great depth. To study the potential effect of changes in agricultural practises on groundwater quality, sampling of subsoil and extracting soil moisture by centrifugation may overcome the problem of lag time. The relative centrifugal force (RCF) rather than the time of centrifugation has major effect on the soil moisture recovery (SMR). The maximum SMR for silt loams at field capacity is about 40%. For high RCFs (above 6600*g*) and centrifugation times above half an hour, the effect of an increase in RCF or time of centrifugation on SMR is small. SMR also depends on clay and soil moisture content, i.e. factors that, with others, determine the actual capillary pressure. Concentrations of most solutes are fairly constant with an increasing SMR, as most solutes, including nitrate, did not show a change in concentrations with an increasing SMR from 5% up to the maximum that can be realised of about 40%.

Drainage centrifugation is a useful tool in the case of a monitoring approach based on statistical sampling where leaching at a large number of sites needs to be monitored and groundwater levels are a great depth. Also in projects where root zone leaching is studied at field level, but do not need continuous monitoring of soil moisture quality, this tool should be considered.
